# Continuity of CVD treatment during the COVID-19 pandemic: evidence from East Java, Indonesia

**DOI:** 10.1186/s40545-022-00509-w

**Published:** 2023-03-22

**Authors:** Aksari Dewi, Elizabeth Pisani, Bachtiar Rifai Pratita Ihsan, Ayuk Lawuningtyas Hariadini, Anushka Patel, Anna Palagyi, Devarsetty Praveen, Sujarwoto, Diana Lyrawati

**Affiliations:** 1 The George Institute for Global Health, University of New South Wales, Sydney, New South Wales, Australia; 2 Department of Pharmacy, Faculty of Medicine, Brawijaya University, Malang, East Java Indonesia; 3 Department of Public Administration, Brawijaya University, Malang, East Java Indonesia; 4 The George Institute for Global Health, University of New South Wales, Hyderabad, India

## Abstract

**Background:**

In Indonesia, the world's fourth most populous country, cardiovascular diseases (CVDs) are a leading cause of death and disability. Government efforts to reduce the burden of CVD include a community-based prevention and early detection programme, and the provision of medicines to prevent cardiovascular events. Disruptions to medicine supply chains, service provision, and movement during the COVID-19 pandemic potentially threatened the continuity of these efforts. We investigated the distribution and dispensing of common CVD medicines in Malang district, East Java, before the pandemic and early in its course.

**Methods:**

From January to October 2020, we collected monthly data on stock levels, sales or dispensing volumes, and price for five common CVD medicines (amlodipine, captopril, furosemide, glibenclamide and simvastatin), from a public and a private distributor, and from public health facilities (*n* = 4) and private pharmacies (*n* = 2). We further complied monthly data on patient numbers in two primary health centres. We tracked changes in stocks held and volumes dispensed by medicine type and sector, comparing the three months before the local COVID-19 response was mobilised with the subsequent seven months. We conducted interviews with pharmacists (*n* = 12), community health workers (*n* = 2) and a supply chain logistics manager to investigate the reasons for observed changes, and to learn details of any impacts or mitigation measures.

**Results:**

The pandemic affected demand more than supply, causing medicine stocks to rise. Restricted service provision, lock-down measures and fear of infection contributed to a sharp drop in patient numbers and dispensing volumes in the public sector. Meanwhile private sector sales, especially of lower-priced CVD medicines, rose. Community health workers attributed some poor health outcomes to interruption in regular patient check-ups; this interruption was aggravated by formal mitigation policies.

**Conclusions:**

Fears that COVID-19 would interrupt medicine availability were unfounded in East Java. Public sector patients may have compensated for reduced service access by switching to private pharmacies. Mitigation policies that ignored administrative procedures were not effective.

**Supplementary Information:**

The online version contains supplementary material available at 10.1186/s40545-022-00509-w.

## Background

In Indonesia, the world's fourth most populous country, cardiovascular diseases (CVDs) account for about a third of all deaths; they are also a leading cause of disability [1]. In an attempt to reduce the burden, the Ministry of Health has since 2012 supported a prevention and early detection programme known as Posbindu PTM. Community health workers (known locally as *kaders*) aim to screen citizens for risk of CVD regularly. Those at low risk may be given advice on maintaining healthy lifestyles, while potentially high-risk patients are referred to primary health centres or private clinics for further clinical assessment. Health facilities may prescribe medication to prevent cardiovascular events, and/or refer on to hospitals for further management [2]. Members of Indonesia's nation-wide health insurance system *Jaminan Kesehatan Nasional* or JKN (which at the start of 2021 covered 222.5 million people) are entitled to free medication [3].

In late 2019, the COVID-19 pandemic emerged in Wuhan, which is the second largest producer of pharmaceutical ingredients in China. The pandemic disrupted global supply chains, including of medicines [4]. Most common essential medicines in Indonesia are produced domestically, but over 90% of active ingredients are still imported; two-thirds of the imports are from China, and a further 17% from India, according to the Indonesian Pharmaceutical Companies Association. The industry grouping feared that pandemic-induced interruption of supplies of active ingredients could potentially interrupt supplies of essential medicines to patients in Indonesia [5].

After Indonesia reported its first case of COVID-19 in March 2020, the government imposed restrictions, including a partial lock-down, to control the spread of the virus [6]. As a result, extension health services were closed, opening hours at primary health centres were restricted, and retail activity was curtailed. All of these measures potentially restricted patients' access to health care, including medicines. This is especially challenging for the management of chronic diseases such as CVDs, which require both regular interface with health staff, and uninterrupted access to therapy, in particular those that lower blood pressure and cholesterol. Underlying CVD also increases the risk of severe illness or death for patients with COVID-19 [7]. In October 2020, the Ministry of Health announced that 13.2 percent of Indonesians reported dead with COVID-19 had hypertension, the highest of any co-morbid condition [8]. This adds to the urgency of keeping CVD under control during the pandemic.

Reductions of supply of essential medicines in low- and middle-income countries during the COVID-19 pandemic have been reported at the global level [4]. However, because limited information was available from the demand side, it was unclear whether changes in supply in fact resulted in shortages. Research into changes in demand has focused mainly on medicines that were believed, sometimes erroneously, to be helpful in combatting COVID-19, including azithromycin, hydroxychloroquine, antivirals and medicines used in intensive care and palliative care [9–11].

This study aimed to examine the effect of the COVID-19 pandemic on the continuity of provision of CVD-related services in health facilities and the availability of medicines in the supply chain, using data from a densely populated rural area in Indonesia.

## Methods

### Study setting and medicines pathways

The research was carried out in Malang District, East Java, Indonesia. The District Health Authority is currently scaling up an enhanced version of the Posbindu PTM programme, using technology to assist community health workers, nurses and doctors in the screening and management of CVD [12, 13].

**Fig. 1:**
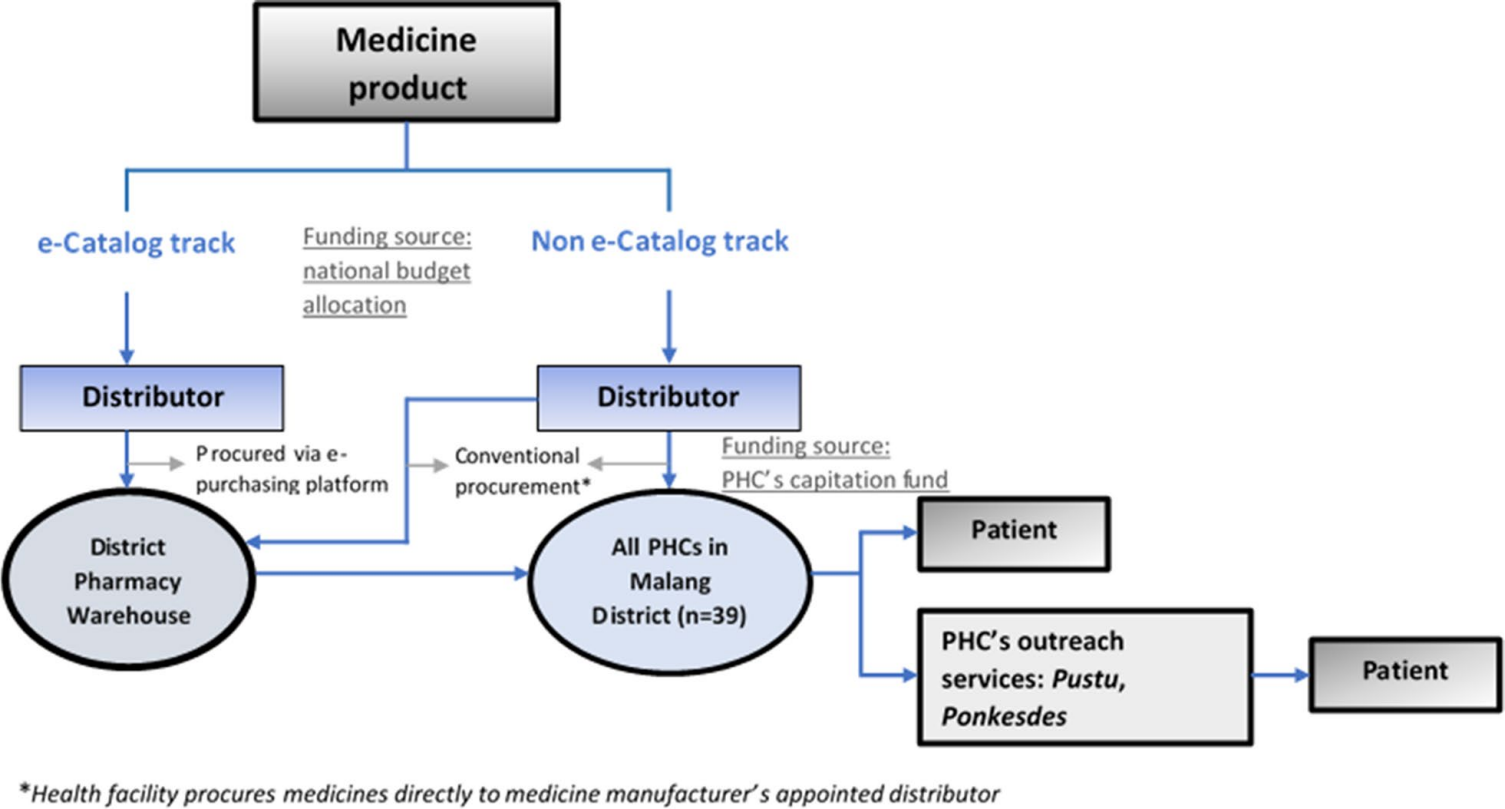
Flow of medicines in the primary health care system

Figure 1 shows medicine distribution in the public primary health care sector in Malang District. The District Warehouse for pharmaceuticals (which is part of the District Health Authority) aggregates annual demand from all primary health care services, and uses budget allocated by the district to procure medicines through the national procurement platform, known as e-catalogue. If there is a shortage of e-catalogue drugs, for example because there were no bidders, or winning bidders fail to supply medicines, the warehouse may procure on the open market. The district warehouse then distributes the medicines to the 39 primary health centres (*Puskesmas*) in Malang District. The distribution volume is based on *Puskesmas* requests, submitted monthly. If the District Warehouse is unable to fulfil a request from a *Puskesmas*, the facility may procure medicines directly, but it must use its own funds, derived from capitation for patients with national health insurance (JKN) coverage.

*Puskesmas* dispense to patients directly via their pharmacy department, but also distribute medicines to their dependent extension services (auxiliary heath centres known as *Pustu* and village health posts known as *Ponkesdes* which provide medicines to outreach programme for non-communicable disease in their coverage villages, known as *Posbindu PTM*)*.* In normal times, cardiovascular patients whose condition is stable go for monthly check-ups at a *Puskesmas* or its satellite extension services; they are given up to a month's supply of medicines, free of charge.

If a patient's condition is severe, they are referred to a secondary hospital facility for treatment. They are transferred back to primary care services for long-term management once their condition is stable. Public hospitals in Indonesia provide medicines free to patients with JKN coverage, paying for them out of a flat fee for patient services reimbursed by the insurer against a diagnostic code. The hospital may procure directly from the e-catalogue platform (often the cheapest option) but may also procure directly from other sources. Patients who are not covered by JKN must pay for their medicines [14].

In the private sector, manufacturers generally appoint a national distributor, which sells medicines to hospitals and pharmacies, often through local distributors. Some pharmacies are reimbursed by the public insurer for providing medicines for chronic conditions to JKN patients, (though none was included in our study). In addition, patients may buy medicines from pharmacies, regardless of their insurance status. Technically, common CVD medicines require prescriptions. In practice, however, many pharmacies sell them without prescription [15]. Some private pharmacies also offer screening services such as cholesterol and blood pressure tests, at a cost to the patient [16].

### Participant recruitment and consent

This study was nested in a larger study of the quality of cardiovascular medicines in Malang District, involving the collection of medicine distribution and price data, and in-depth interviews with individuals with either direct experience, or knowledge of, the landscape relating to CVD medicines in Malang, including funding, pricing, procurement, distribution, prescription, dispensing and record-keeping [17]. Potential participants were approached by e-mail outlining details of the study and requesting their participation; those who agreed to participate provided verbal or written consent, including for the recording of interviews. A sub-set of participants who consented to participate in these in-depth interviews were asked if they would consider participating in the current sub-study to track developments in the supply chain that might result from the COVID-19 outbreak, by providing monthly quantitative data and short comments about any unusual supply or demand-related issues that emerged over the study period. For this sub-study, we aimed to included participants representing each of the following supply chain actors: the District Warehouse, the district hospital, a *Puskesmas*, a community health worker, a medicine distributor, a chain pharmacy and an independent pharmacy. Those who expressed an interest in participating were re-contacted with further details about the monthly data collection; participants also provided separate verbal consent for this sub-study.

The study received ethics approval from the researchers' home institutions, in Indonesia and another country [details on cover page as requested for blinding].

### Data collection

#### Quantitative monthly data collection

For the monthly data, we provided each participant with a standardised format of Excel spreadsheet to record data on the five most frequently prescribed essential medicines for CVD risk management in the study area. We chose the study medicines based on a 2018 household survey data, in which over 6,500 high-risk patients reported which (if any) medicines they took to control blood pressure or cholesterol [12].We included all medicines and dosages taken by at least 10% of those reporting medicine use. Because of high levels of co-morbidity, these included one medicine (glibenclamide) to control blood sugar. In order of frequency the medicines were amlodipine, simvastatin, captopril, furosemide and glibenclamide, all in oral tablets. The first three are commonly prescribed in two dosages, the final two in just one, giving a total of eight products (APIs and doses). The required information variables are arranged as in column 1 of Table 1. The Excel spreadsheet format was standardised to obtain uniform data. We also accepted data in other formats (facility reporting forms, procurement system printouts, photos of stock cards) according to the participant’s preference. Table 1 shows the information collected. Data were collected separately for each medicine, dosage and brand.

**Table 1:** Data collected monthly, by facility

Information	Facility				
	Warehouse	Distributor	Pharmacies	*Puskesmas*	Hospital
Current stock	X	X	X	X	
New stock in	X	X	X	X	
Stock distributed	X				
Stock sold		X	X		X
Stock dispensed for free				X	X
Acquisition price	X				
Sales price		X	X		X
Number of patient visits				X	

Prospective data collection began in March 2020, and continued until October 2020. We also requested retrospective data from January 2020. The first COVID-19 case in Malang was confirmed on March 18, 2020 [18, 19], and some restrictions on services were introduced by March 24th. Because our data were reported for whole months, in our analysis we compared volumes of medicines distributed or dispensed from January to March (the "pre-pandemic period") with volumes distributed/dispensed over the first seven months of the local pandemic (the "pandemic period", April–October).

#### Qualitative data collection: in-depth interviews

For the in-depth interviews, interviewers used a pre-defined semi-structured interview guide, tailored to the roles and responsibilities of each participant and covering a range of subjects broadly related to the medicine supply chain. The interviews, conducted in Indonesian, were recorded and transcribed verbatim. In-depth interviews were conducted in June and July 2020, relatively early in the COVID-19 pandemic. We conducted follow-up interviews with participants who had consented to provide monthly data, focusing specifically on changes in supply and demand for CVD medicines observed in the monthly data. These were also recorded and transcribed. Participants providing monthly data also sometimes provided short comments to a member of the research team highlighting unusual supply or demand issues, usually by electronic message. These were collated. To reduce the risk of COVID-19 transmission, most interviews were conducted remotely by telephone, or using the WhatsApp or Google Meet platforms.

### Data management and analysis

Quantitative data were entered into a database, with one record per medicine type, dose, brand, facility and month, resulting in 1,454 observations. Descriptive analyses were conducted using STATA 17. We analysed trends in dispensing by price of medicines to the patient, differentiating between those provided for free in the public system, and those sold at premium and non-premium prices. We defined premium medicines as those priced at over 1.5 times the local market median for the medicine and dosage. When comparing trends among medicines and outlets with very different volumes, we described variations relative to the reference period of January 2020. For example, if sales were 65% higher in February than January, the February index value would be 1.65.

We analysed the qualitative data deductively using a framework based on evolving discussion in the news, institutional and eventually the academic media of the putative or measured impact of the COVID-19 pandemic on medicine supply chains or health service provision [11, 20–24].

The framework defined the following areas of interest:

- types of medicines available by outlet;
- changes in availability of stocks at the wholesale/District Warehouse level over the course of the COVID-19 pandemic;
- changes in demand, by type of outlet;
- reasons for any observed or reported changes in supply or demand;
- health impact of any observed or reported changes in supply or demand;
- strategies adopted to mitigate any negative impacts of the COVID-19 pandemic on CVD services.

**Fig. 2:**
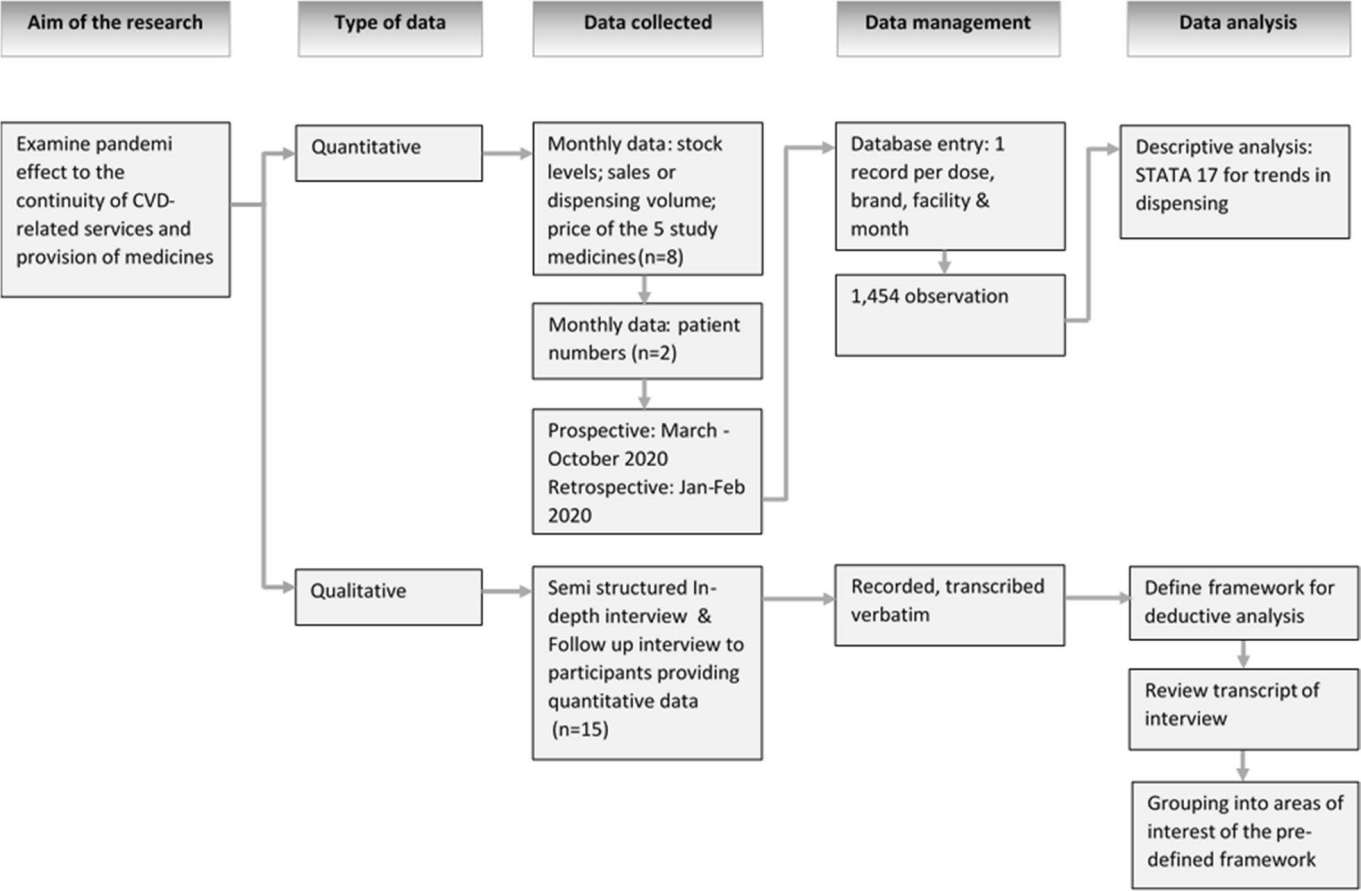
Research procedure

Two authors (AD and EP) reviewed all interview transcripts for information related to the continuity of service provision (especially medicine supply) for CVD patients, grouping them into areas of interest. They determined through discussion the extent to which responses differed between facilities, or explained, supported or diverged from changes in supply or demand observed in the quantitative data [25]. Figure 2 describes the research procedure in this study.

## Results

Pharmacists or logistic staff in eight facilities provided monthly data, and 15 respondents participated in in-depth interviews. Twelve interviewees were pharmacists, one was a logistics manager and two were community health workers. Table 2 shows the details of respondents by sector.

**Table 2:** Monthly data collection and in-depth interview contributors

Contributor	Sector	Monthly data (facilities)	In-depth interviews (individuals)
District warehouse	Public	1	2
Primary health centre (*Puskesmas*)	Public	3	4
Posbindu PTM	Public	0	2
District hospital	Public	1	1
Local medicine distributor	Private	1	1
Independent pharmacy	Private	1	3
National chain pharmacy	Private	1	2

Several types of facilities are unique in their category, so issues of saturation did not arise in those cases. We collected data from 3 of 39 *Puskesmas* in the study area. We collected monthly data from just two of the 213 private pharmacies reported to exist across Malang district, while pharmacists at another two were interviewed early in the pandemic [26].

One of the three *Puskesmas* did not provide monthly data for medicines purchased with capitation funds. The community health workers did not provide monthly dispensing data because outreach services were closed.

### Medicines profile

**Fig. 3:**
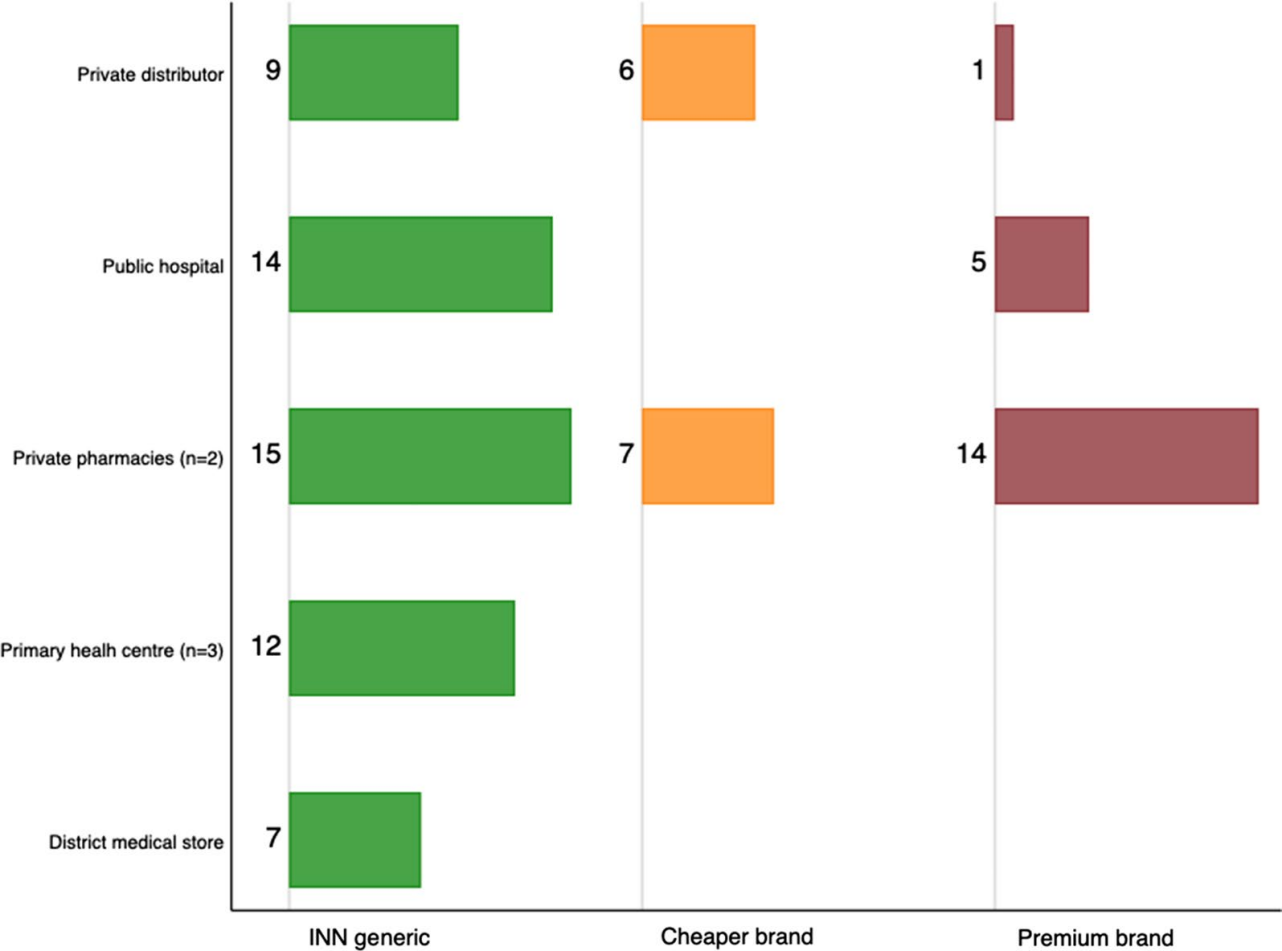
Number of study medicines stocked by various outlets, by brand status

National regulations dictate that all off-patent medicines provided in the public system should be unbranded International Non-proprietary Name (INN) generics [27]. In practice, we found the public hospital made branded generics available to patients not covered by JKN. Patients who are unable or unwilling to use JKN may also choose to buy INN or branded medicines at private pharmacies. This is reflected in Fig. 3, which shows the brand profile status in the public and private sectors.

Staff in private sector pharmacies reported in interviews that they selected their brand mix according to their target clientele. Branches of national chain pharmacies often stock premium products; this was the case in our study. The independent pharmacy that provided us with monthly data, on the other hand, favoured INN products and non-premium brands.

### Medicines flow and dynamic during the time of observation

#### Availability of medicines for distribution

**Fig. 4:**
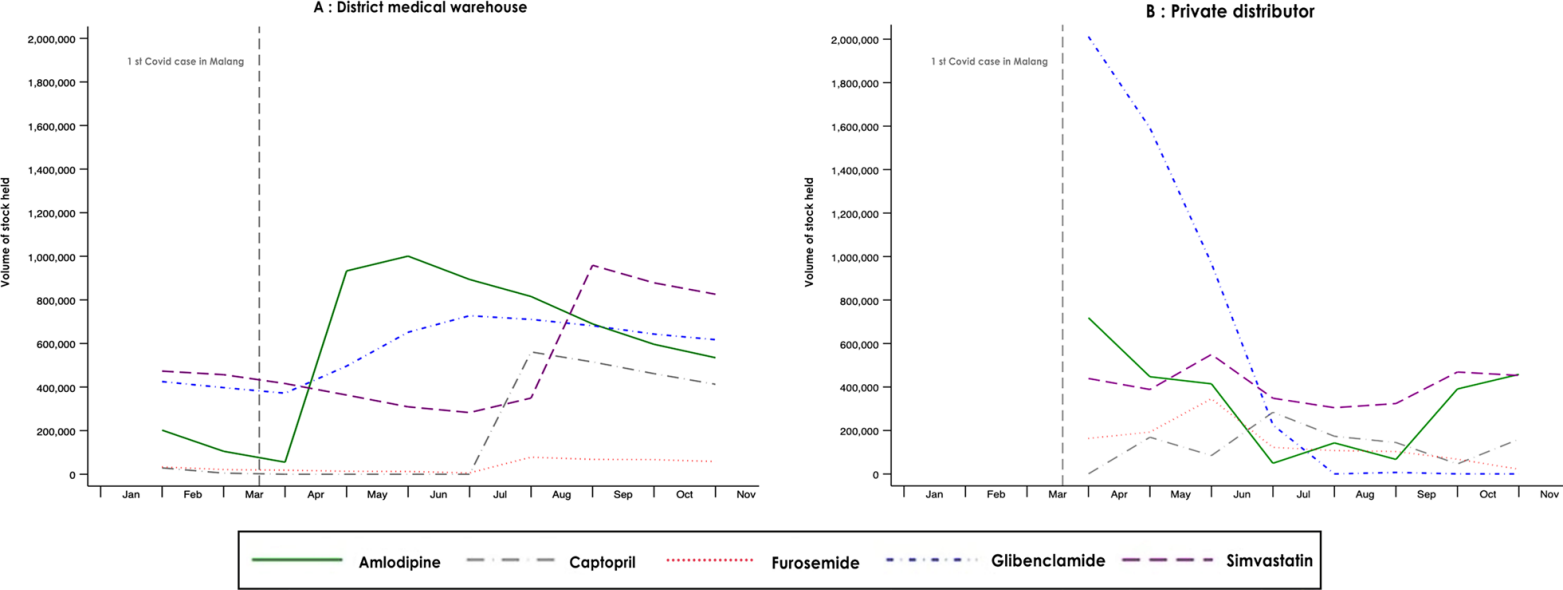
Change in stock held by the District Warehouse and a private distributor of 5 CVD medicines, January–October 2020

The District Warehouse reported stocks end of each month from January to October; Fig. 4A shows stocks in hand at the start of each month from February. A local private distributor was unable to provide data for the first quarter; Fig. 4B shows data on stocks available from the start of April.

Stocks of most medicines remained available throughout the first 7 months of the pandemic. In an interview, District Warehouse staff explained that although supplies of personal protective equipment were interrupted, pre-pandemic orders for medicines by the warehouse continued to be fulfilled. Since demand from health centres for amlodipine and simvastatin, the two most commonly prescribed medicines, fell over the study period, this led to an overall build-up of stocks of CVD medicines compared with the pre-pandemic period. At the end of March, the month the first case of COVID was reported in Malang, the Malang District Warehouse held a total of 861,740 pills across all five medicines. Stocks rose to peak at 2,666,220 by the end of September, falling slightly to 2,475,280 pills by the end of October, still 187% over the end-March level.

The exception in terms of availability was captopril, which was out of stock at the District Warehouse from May to July 2020. Interviewees explained that the public procurement auction for captopril, which predated the COVID-19 epidemic, failed for the delivery year of 2020, so no captopril was available from e-catalogue. The warehouse's 2019 stocks lasted until April 2020. The warehouse was then able to procure from other sources for delivery in July.

#### Changes in demand: fewer patients seeking care

**Fig. 5:**
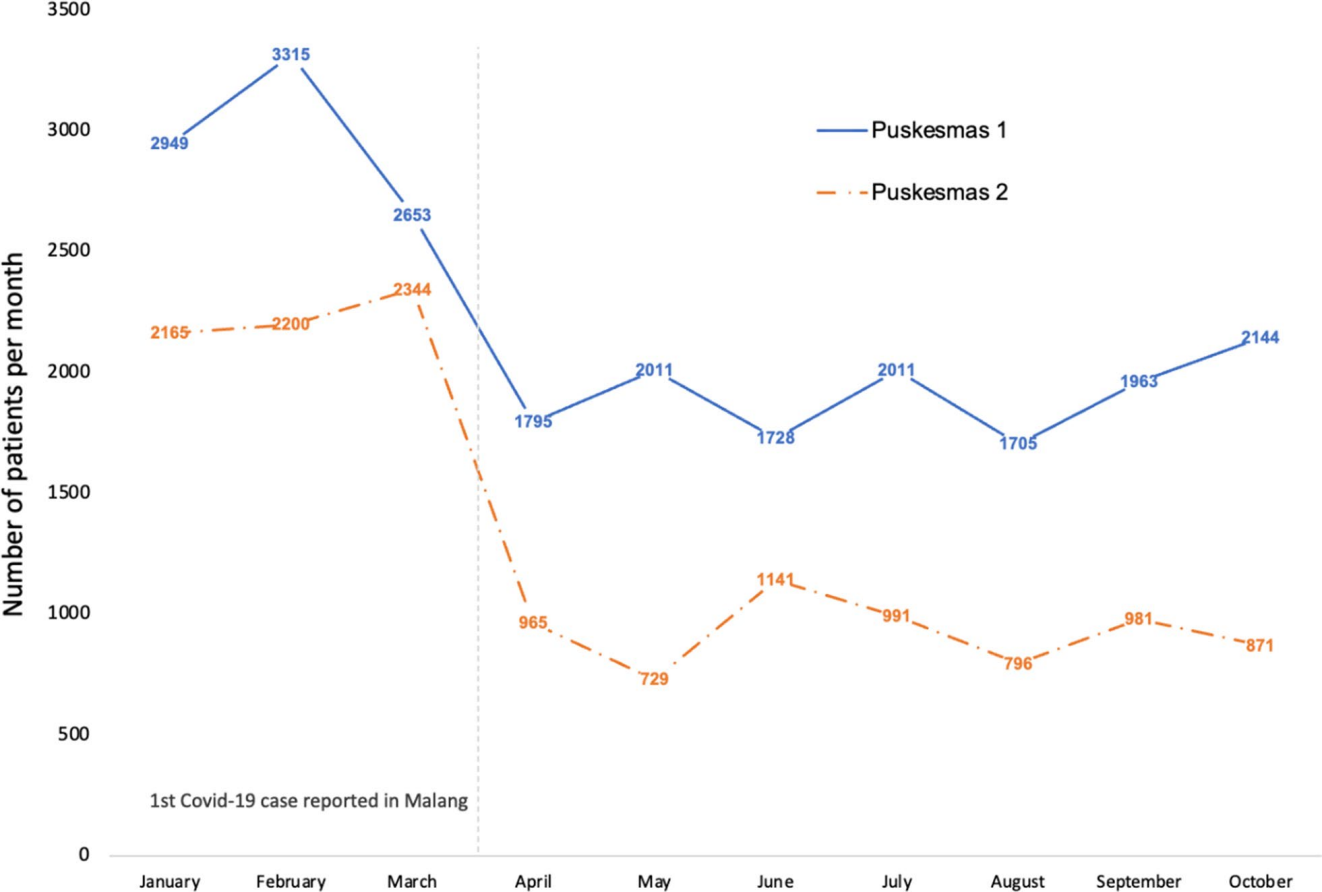
Outpatient visits to two primary health centres, January to October 2020

All three participants representing *Puskesmas* reported a significant reduction in patient visits to their facility in the early period of the pandemic (disaggregated data by presenting condition was unavailable). From March 24th 2020, and for the duration of the study, patient registration counters opened for just 3.5 h a day, down from 5 h pre-pandemic, limiting the number of patients who could be seen. As Fig. 5 shows, outpatient visits at *Puskesmas* 1 began falling in March 2020, then tended to stabilise at lower levels from April. Puskesmas 2 recorded a steep decline in patient numbers between March and April 2020, with no real recovery of patient numbers by October.

The third *Puskemas* did not provide patient numbers by month, but also reported a significant reduction in patient visits:

“*Before COVID, we used to get around 140 patients on Mondays and maybe 100 to 110 patients on the other days. After the outbreak we limited service hours. The counter closes at 10.30 in the morning where before it closed at noon. So numbers have fallen to around 75 – 80 patients on Monday, and 40 – 50 on the other days*”—harmacist, Puskesmas 3.

The public hospital’s pharmacist, who similarly reported a decrease in patient visits due to the pandemic, attributed this decline to patients' fear of being exposed to the virus if they visited the hospital, which was a referral health facility for COVID-19 treatment.

### Changes in dispensing

**Fig. 6:**
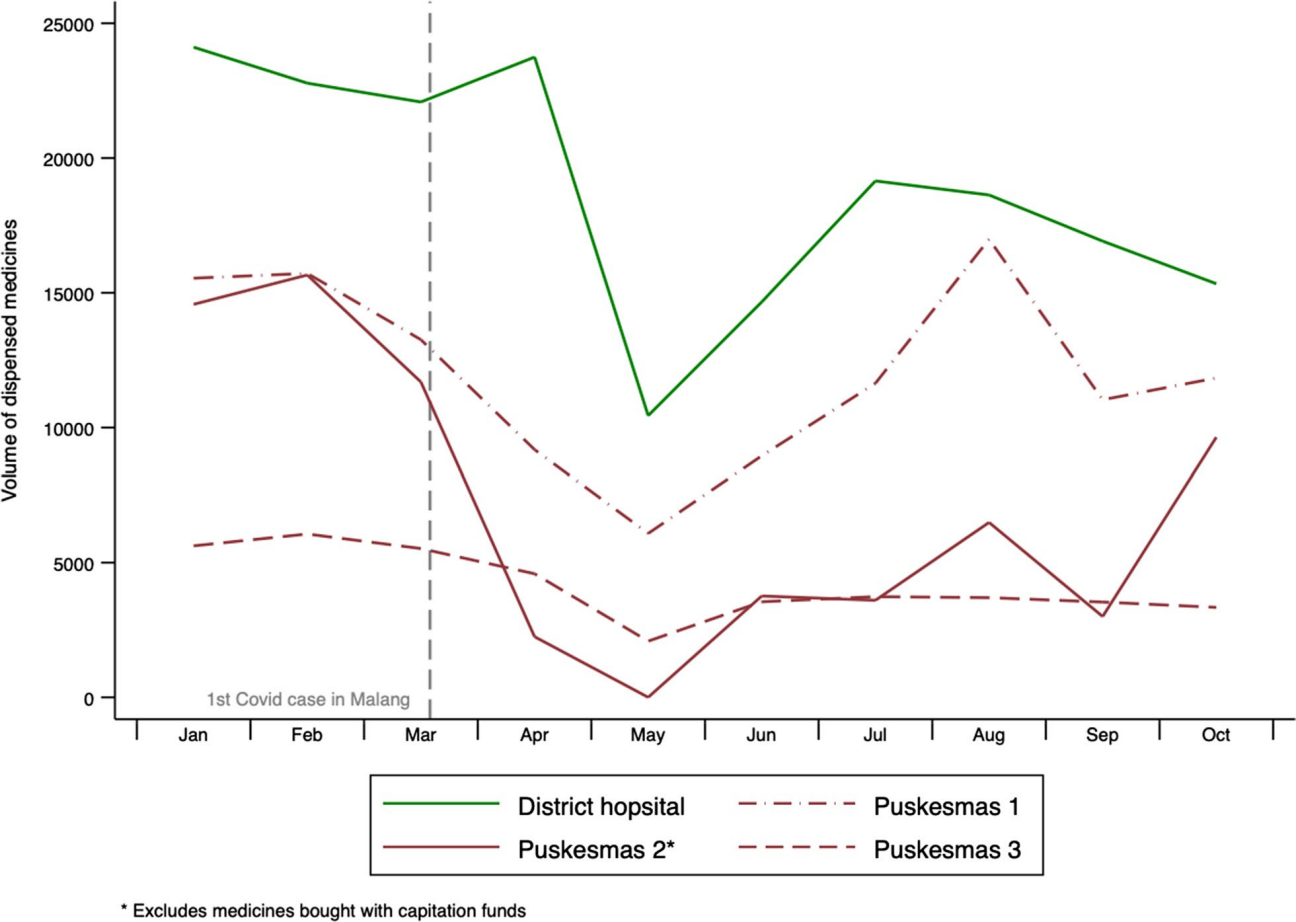
Absolute volume of CVD medicines dispensed at the district hospital and participating Puskesmas, by month (January–October 2020)

The medicines dispensing data shown in Fig. 6 were consistent with the reduction in visits from CVD patients reported by the public sector pharmacists. From January to March 2020, the hospital dispensed a monthly average of 22,880 units (pills) of the five study medicines. April dispensing levels were similar to pre-pandemic levels, but in May 2020, dispensing dropped by 56% (against April) to 10,441 units. Between May and October 2020, average monthly dispensing was 15,855 units. Comparing January–March with April–October (pre-pandemic with pandemic periods), hospital dispensing was down 26%.

As Fig. 5 shows, the dispensing pattern at all three study *Puskemas* was similar. From a peak of 37,439 units dispensed in February 2020, volume fell by 78% to a low of 8,166 in May 2020. Monthly dispensing averaged 18,424 units over the study’s ‘pandemic period’, 50% lower than the 36,586 average recorded in the January–March period.

**Fig. 7:**
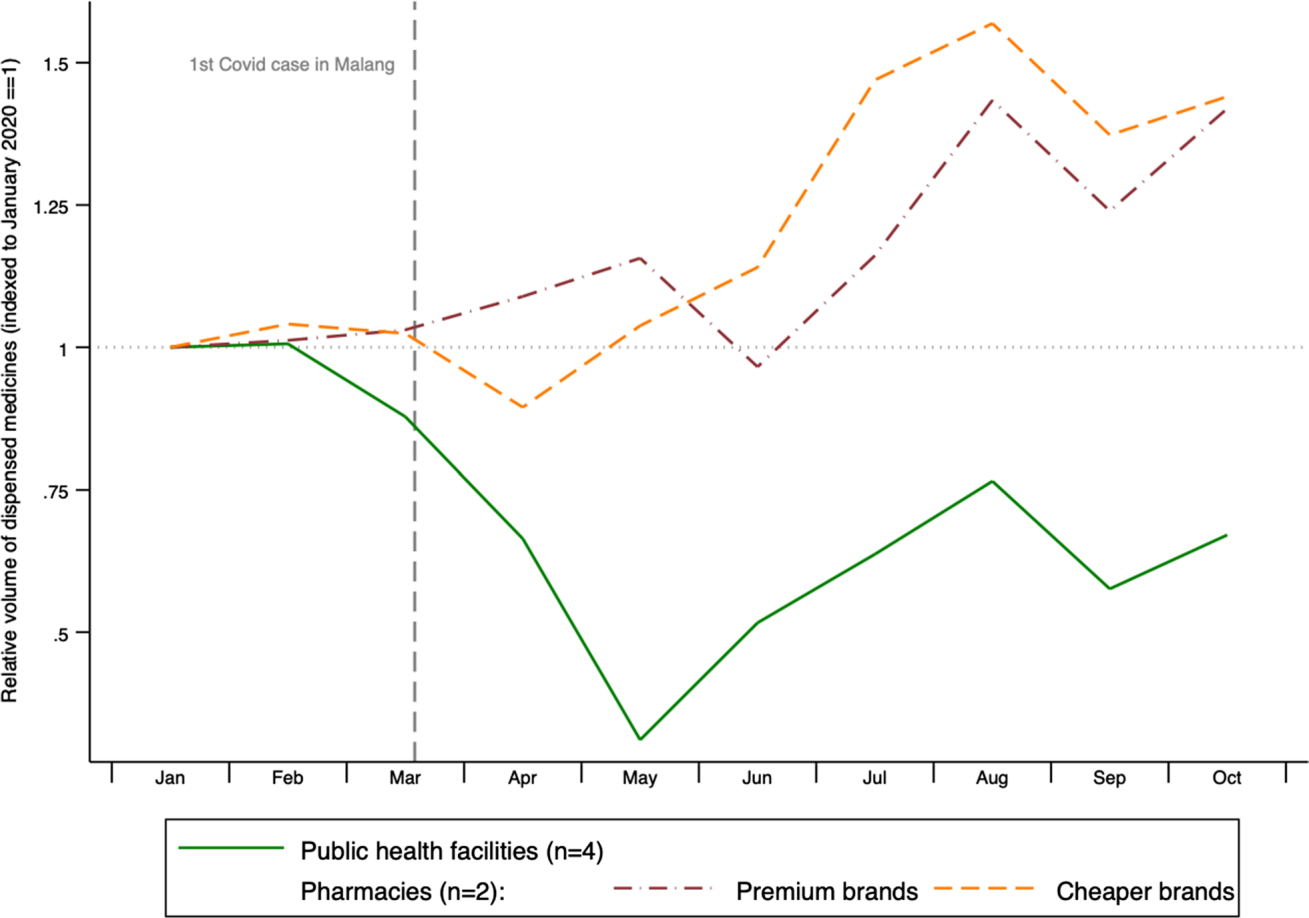
Relative change in monthly dispensing of 5 cardiovascular medicines, January–October 2020, by sector and price of medicine

Figure 7 combined the data shown in Fig. 6 to compare dispensing in the public sector with that in the two private pharmacies in our study, the latter further disaggregated by the type of medicines they sold (premium and cheaper brands). Monthly dispensing volumes are shown relative to their value in January 2020.

In the private sector pharmacies, sales volume changed little over the first three months of 2020. In April 2020, sales of cheaper brands fell by 12% compared with the January–March average, rising steadily to 53% over their "pre-pandemic" levels by August. In the same month, the volume of premium brands sold reached a high of 39% above pre-pandemic levels. Average monthly volume sales of non-premium (cheaper) brands were 25% higher in the pandemic period compared with in the first three months of 2020, while sales of premium brands rose by an average of 19%. Additional file 1: Fig. S1A, Additional file 2: Fig. S1B, Additional file 3: Fig. S1C, Additional file 4: Fig. S1D, Additional file 5: Fig. S1E show these data by study medicine. The increase in private sales volumes was particularly pronounced for amlodipine, the most widely prescribed of the study medicines (Additional file 1: Fig. S1A). Average monthly sales of cheaper brands of amlodipine rose by 74% in April–October compared with the pre-pandemic period, while premium brands rose by 29%. This compares with a 45% drop in the volume of amlodipine dispensed by the public sector.

Staff in private sector pharmacies (including in pharmacies not providing monthly data) interviewed in June confirmed that client numbers were significantly lower than before the pandemic. Follow-up interviews with those providing monthly data provided insights into the fluctuation in sales.

*“At the beginning of the pandemic we experienced decrease in sales. Mostly because people were afraid to go outside, afraid to go for routine check-ups. And WFH [work from home] seems to have impacted the PBF [distributors] as many of our medicines were out of stock, so we had to turn patients away... But then towards the end of the year sales almost doubled. [Patients] said they were stocking up so they don’t have to go back and forth to the pharmacy”—*Private sector pharmacist

### Health impact

Data on health impacts of the COVID-19 pandemic on CVD patients were limited to insights provided in interviews with health workers in the community. A health worker who supported *Puskesmas* outreach health programmes delivered through *Posbindu PTM* (prior to the suspension of these services in March 2020) described the effect of the lockdown.

Interviewer: “When the Posbindu PTM re-opened in September, how were the health measurements for most of the patients?”.

Respondent: “They got worse, especially for hypertension. The blood pressure measurements were elevated in almost all patients because they just didn’t take their medicines regularly. They might have got their medicines elsewhere [i.e. not from the Posbindu PTM], but the adherence level is pretty low.”

Interviewer: “Did you hear of any patients being hospitalised or dying as a result?”.

Respondent: “Maybe not dying. But [patients] hospitalised, yes. Because like I told you, some patients live by themselves at home, no one reminded them [to take the medicines], and then finally they had a stroke. We have those cases.”

Community health worker 1, *Posbindu PTM*.

A second community health worker said that patient adherence was often only partial. Lack of outreach services during the pandemic meant that patients were not reminded to take medicines, even when their blood sugar levels rose, putting them at increased risk.

Since [the pandemic] began, people rarely got checked. Ok, the adherence level is never 100%, but let's say its 80% that still increases your life expectancy, right? The way I see it, it's still better for them to be taking their medicine, even at only 60%, rather than not take it at all. If they don't take it at all, they'll leave us [die] sooner. If they get checked monthly, I can say "Look, Granny, your sugar's up a bit," and then she goes back to taking her medicine."

—Community health worker 2, Posbindu PTM.

### Policy and implementation responses

From early in the pandemic, Indonesian authorities and health personnel were well aware of the importance of avoiding interruptions to services for patients with chronic conditions. BPJS Kesehatan, the agency which manages the JKN, thus made adjustments to keep services on track. One, known as the "iteration" programme, was issued on 22 March 2020. It allows chronic disease patients to re-use a 30-day prescription, returning after a month to claim medicines for a second month without getting a check-up [28]. According to a hospital pharmacist, the policy was well-intended but implementation was problematic, in particular since without a doctor's visit, the hospital cannot claim against a diagnostic code, which is supposed to be "packaged" to include the price of medicine.

“*We faced many issues, especially when it came to claiming reimbursement. Most often the referral letter from the primary referrer had expired. Also, some patients stopped paying their premium, so by the second month their JKN membership was inactive. These two administrative issues caused rejection when we submitted the claim to the system. In terms of financing, that really means the hospital is making a loss, because the claim is only for non-packaged medicines, meaning there is no fee for service provision.*”—Head of Pharmacy Department, Public Hospital

The pharmacist also reported many patient complaints. The policy was abandoned at the end of May 2020 after a 2-month trial period.

On the procurement side, there was some evidence that the public sector struggled to respond. Usually, demand planning and budgeting is based primarily on the previous year's data, adjusted for population growth, inflation and other factors. But as a respondent from the District Warehouse pointed out, the unusual patterns of demand in the epidemic (and the residual over-supply) made that difficult.

“*There was a significant reduction in medicine distribution in 2020. That’s why I was so confused when I did the procurement planning for 2021. If I bring down planned volumes to match [2020] data, what if turns out next year [2021] we need more? But if I plan [to order] more [stock] then what happens if the Puskesmas request less? This is such a dilemma. But finally, we decided to order more. Originally we planned less, using 2020 data, but then we got more funds for medicine procurement, so I used 2019 data as reference for the planning.*”—Pharmacist, District Pharmaceutical Warehouse, DHA

## Discussion

Two months after declaring COVID-19 a global pandemic, the World Health Organization expressed concern about continuity of essential health services. The institution carried out a rapid assessment of services for chronic disease management, and found that hypertension management services had been disrupted in over 50% of responding countries [29].

The first COVID-19 case in Malang, a district in Indonesia's second most populous province of East Java, was confirmed on March 18, 2020 [18, 19]. Shortly thereafter, the government imposed restrictions on movement, affecting many aspects of life, including access to health services for patients with chronic conditions, such as those at high risk for cardiovascular disease. Community outreach services (through which doctors provide medicines and monthly monitoring, and extension workers support adherence) were suspended. Service hours at primary health centres were shortened, and transport (including to the district hospital) was curtailed. These access restrictions raised the possibility that patients at high risk for heart disease may interrupt medication taken regularly to control blood pressure and cholesterol in an East Java community where 29% of adults over 40 have high cardiovascular risk [12]. The potential for the pandemic to interrupt the supply of essential medicines, a possibility expressed globally and domestically, heightened concerns that the pandemic may undermine CVD management.

Examining the flow of five cardiovascular medicines before and after the start of the pandemic in Malang district, we found that fears about disruptions to supply were largely unfounded. While studies in countries that are heavily dependent on imported medicines reported interruption of supplies of essential medicines at the national level [30], this was not the case in our study. Although one pharmacist mentioned difficulty in securing deliveries of some essential medicines, primary health centres and private suppliers had supplies of all cardiovascular medicines on hand, and for most study medicines, stocks at the District Warehouse rose over the study period.

We have proxy indicators for consumption, in the form of patient numbers (all conditions) and dispensing data (CVD medications) from four public facilities, and sales data from two private pharmacies. At *Puskesmas*, which restricted service hours, overall patient numbers fell significantly, while at CVD outreach services they dropped to zero until services resumed in September 2020. Dispensing of CVD medicines fell sharply at all public facilities, reaching their lowest levels in May 2020 (which coincided with the fasting month).

While private pharmacies reported an initial fall in dispensing, sales volumes quickly picked up again (including in the fasting month), substantially exceeding pre-pandemic levels within 3 months of the onset of COVID-19 locally. While it is possible that resulted in part from stockpiling by patients who wished to secure supply and had the means to do so, the increase was highest for INN generics and other non-premium brands. This suggests that some patients who would normally get medicines free in the public sector may have been buying low-cost medicines from pharmacies during the pandemic. Unlike health facilities, pharmacies in Malang District do not routinely ask for prescriptions for common CVD medicines. Patients with co-morbidities associated with a high risk of mortality among COVID-19 patients may have preferred to pay for medicines than to queue at health facilities, not least because they can send lower-risk family members to pharmacies, thus reducing their own risk of exposure. Public health warnings that COVID posed a particularly high risk to cardiovascular patients may have motivated patients to continue taking CVD medicines, even when they were unable easily to access them for free. A policy restricting access to COVID-19 vaccination for people with blood pressure exceeding 180/110 mmHg provided an additional incentive not to interrupt treatment for hypertension [31].

On average, citizens of Malang district spend around 958,000 rupiah (USD 67) a month on food and non-food consumption; for the poorest fifth, spending is roughly a third of that. In comparison, a month's supply of INN generic amlodipine costs around 3,270 rupiah (USD 0.23). While INN versions of all the other study medicines are similarly affordable, some patients will inevitably have gone untreated. Pharmacists noted that immunostimulant supplements were widely promoted during the pandemic. This may have encouraged poorer patients to invest their health spending on supplements for the prevention and control of COVID-19, rather than pay for CVD drugs to control conditions which often do not show severe symptoms.

Researchers in Western Kenya also reported challenges in ensuring continuity of medicine supply to CVD patients [32]. In that much more rural setting, and within the context of localised services provided with support from a large on-going academic partnership, managers responded by dispensing medicines for a 90-day period. In Malaysia, dispensing switched to 60 days [33]. While Malang suspended community-based dispensing, the Kenyan and Malaysian programmes sought to increase it, providing pick-up points for medicines in more locations and providing delivery options for patients with limited mobility. Several Indian states initiated community-based dispensing or home delivery of anti-hypertensive medicines by primary care workers. The programme also aimed to dispense medicines for 60 or 90 days rather than the usual 30, although in some states it was hampered by lack of supply [34]. In many other countries, regulators and community pharmacists collaborated to find flexible solutions which allow patients to continue to access medicines for chronic conditions despite restrictions on movement [35].

In Indonesia, the response has so far been mostly at the level of a public insurer covering a population of 222 million people. In March 2020, the insurer proactively announced changes to procedures in response to the pandemic. For chronic conditions, this was centred principally on allowing patients to come back for a prescription refill without a physical consultation with a doctor. This differs from the approach of the countries cited above, which did not require patients to return to hospitals or pharmacies for monthly refills; it was followed by a sharp drop in dispensing at the district hospital. Dissatisfied patients may have chosen to buy medicines at community pharmacies rather than risk going back to the hospital—a referral centre for COVID-19 patients—without even the benefit of a check-up. The programme was abandoned after the trial period, following protests from hospitals about its implementation, including the difficulty in getting reimbursed reported in our study.

As well as a stable supply of medication, effective control of CVD requires regular monitoring of a patient's clinical condition. Many private pharmacies in the study areas do offer reasonably priced instant blood pressure, cholesterol, and blood sugar measurement services to patients who come in to buy their own medicines, providing some indication if therapies are not working. However clinical knowledge and judgement are required to adjust medication in response to risk levels; if patients are unable or unwilling to visit health facilities, their treatment may suffer.

A common response to the challenge of maintaining care for chronic disease patients during the pandemic in countries at all income levels has been to promote greater use of telemedicine [35–37]. A study in Pakistan suggests that community pharmacists can play a vital role in disease management in the COVID-19 era by providing triaging and basic consultations via Telemedicine, whilst doctors, nurses, and paramedics are physically present in the emergency, isolation wards and quarantine centres. This can greatly reduce the burden off the doctors and health system [38]. Although technology-assisted CVD risk screening in the community is being integrated into routine health services in Malang, telemedicine was not integrated into public sector service provision for high-risk patients during the time of our study. However, the potential to increase digital support for patients exists. Indonesia has one of the world's highest penetrations of mobile phones, and village based community health workers in this area are already trained in the use of digital-assisted outreach support [12, 13, 39].

We note that medicine stocks at the District Warehouse rose as demand from public facilities fell. At the same time, private distributors shifted stock more quickly than usual. While one pharmacist said some distributors were out of stock, the distributor included in our study was able to resupply quickly. The difference illustrates the risk of planning procurement orders annually or bi-annually—a common practice in public procurement [40]. Where flexibility to respond rapidly is limited, dramatic changes in demand can lead to shortages or over-supply, while the ability to predict future needs is constrained.

### Strengths and limitations

This study was nested into a larger study of medicine quality planned before the COVID-19 pandemic began. This allowed us to rapidly propose (and get ethical approval for) additional data collection to track supply and demand for CVD medicines in real time. We were able to use interviews to provide context and explanatory data to help interpret trends in service provision and medicine distribution and dispensing. Our study would have benefited from more complete data on numbers of cardiovascular patients at health services, and the participation of more private pharmacies. We were also not able to speak to patients or physicians, or to collect data on clinical outcomes. Though community health workers reported that cardiovascular patients suffered as a result of the pandemic, we were unable to assess the health impact of any COVID-19-related changes in access to or use of CVD services in the study area.

## Conclusion

Our study suggests that, in the Indonesian context, the private sector was able to pick up some of shortfall in CVD medicine provision reported by public services, including increasing its sales of INN and other affordable generic medicines. Patient monitoring, however, may have suffered more. Expanding the role of technologies already in use for risk screening in Malang and similar settings may help to fill this gap.

In addition, we found that national-level responses designed to avoid interruption of treatment for chronic diseases did not immediately fulfil their aims. This underlines the importance of considering administrative burden, feasibility issues and perverse incentives at the implementation level when responding to challenges such as a pandemic; of consulting service providers when planning changes that affect them; and of providing implementation guidelines with room for risk mitigation.

## Supplementary Information

**Additional file 1: Fig. S1. A.** Relative change in monthly dispensing of Amlodipine, January–October 2020, by sector and price of medicine.

**Additional file 2: Fig. S1. B.** Relative change in monthly dispensing of captopril, January–October 2020, by sector and price of medicine.

**Additional file 3: Fig. S1. C.** Relative change in monthly dispensing of Furosemide, January - October 2020, by sector and price of Click.

**Additional file 4: Fig. 1. D.** Relative change in monthly dispensing of glibenclamide, January–October 2020, by sector and price of.

**Additional file 5: Fig. S1. E.** Relative change in monthly dispensing of simvastatin, January–October 2020, by sector and price of medicine.

## Data Availability

Data on stock and distribution volumes over time by molecule, doseform, INN status, price bracket, outlet type (distributor, health facility, pharmacy) can be accessed at https://doi.org/10.7910/DVN/38AIJO, under a CC0 license. Supplementary figures uploaded to the editorial management system are additionally available using the same doi.

## Abbreviations

API: Active Pharmaceutical Ingredient
COVID-19: Coronavirus disease of 2019
CVDs: Cardiovascular diseases
District Warehouse: District warehouse for pharmaceuticals
IDR: Indonesian Rupiah
INN: International Non-proprietary Name
JKN: Jaminan Kesehatan Nasional: national health insurance
PBF: Pedagang Besar Farmasi: medicines' private distributor
*Ponkesdes*: Pondok kesehatan desa: village health posts
*Posbindu* PTM: Pos Pembinaan Terpadu Penyakit Tidak Menular: The Indonesian Ministry of Health's programme for prevention and early detection of non-Communicable diseases
*Puskesmas*: Primary health centre
*Pustu*: Puskesmas pembantu: auxiliary heath centres
WFH: Work from home
