# Supplementary figures and images for: Continuity of CVD treatment during the COVID-19 pandemic: evidence from East Java, Indonesia
Source: J Pharm Policy Pract. 2023 Mar 22;16:50. doi: 10.1186/s40545-022-00509-w (PMC10032625; doi:10.1186/s40545-022-00509-w)

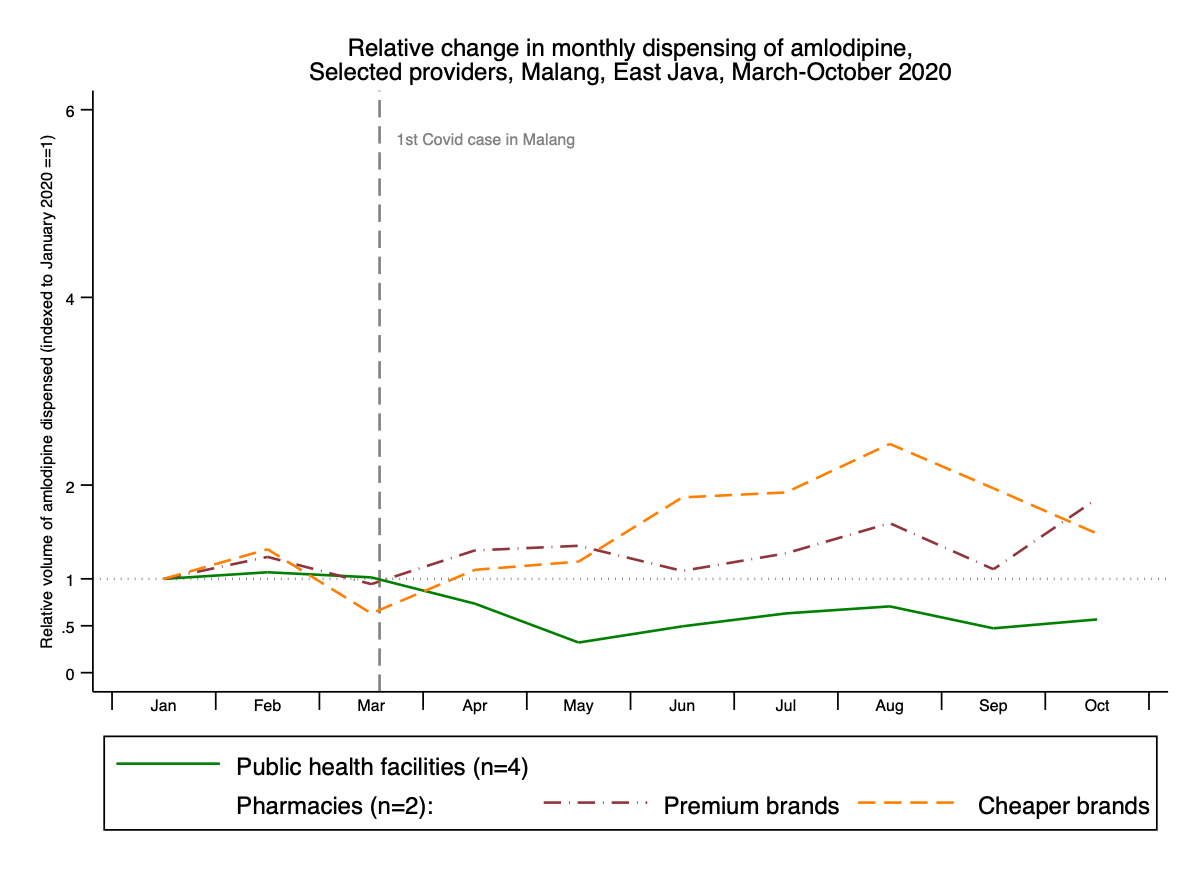

Supplement: Supplementary file 1 — Additional file 1: Fig. S1. A. Relative change in monthly dispensing of Amlodipine, January–October 2020, by sector and price of medicine. [file 40545_2022_509_MOESM1_ESM.png]

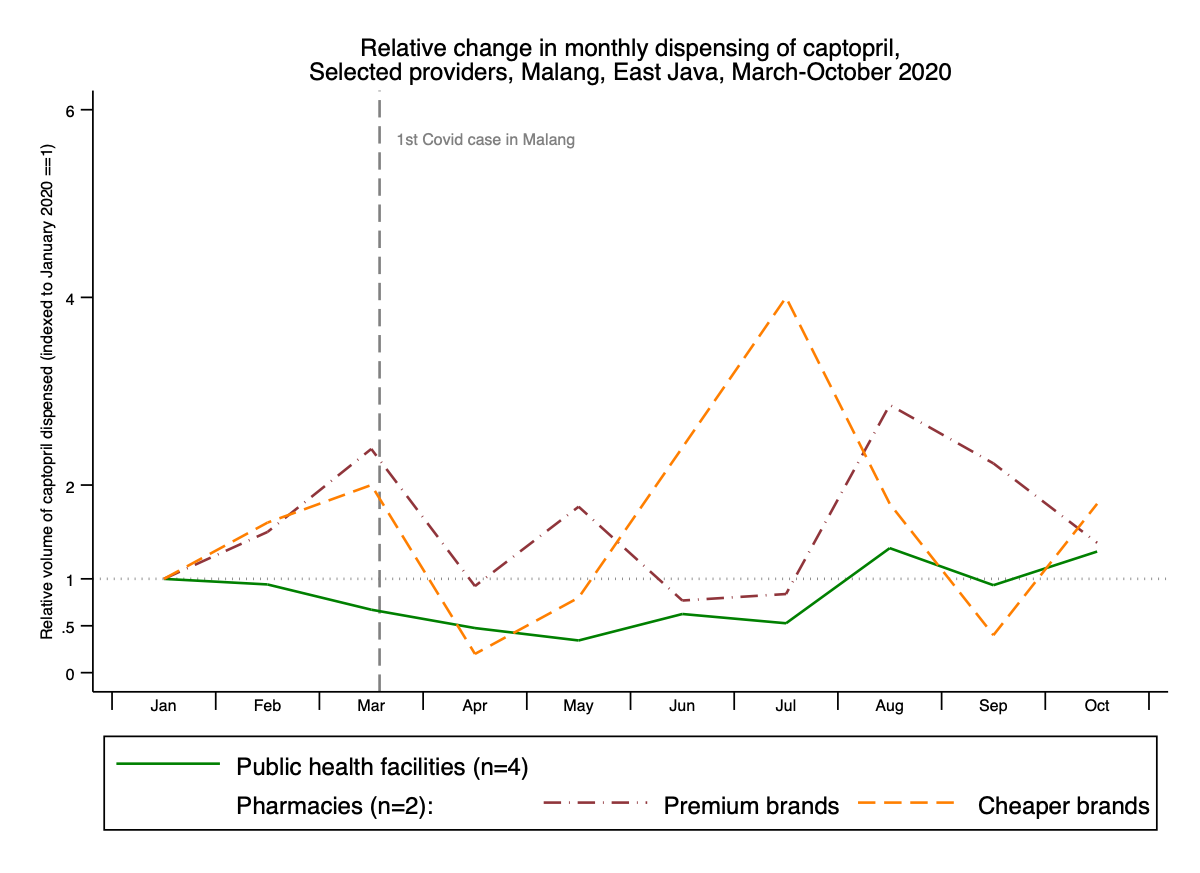

Supplement: Supplementary file 2 — Additional file 2: Fig. S1. B. Relative change in monthly dispensing of captopril, January–October 2020, by sector and price of medicine. [file 40545_2022_509_MOESM2_ESM.png]

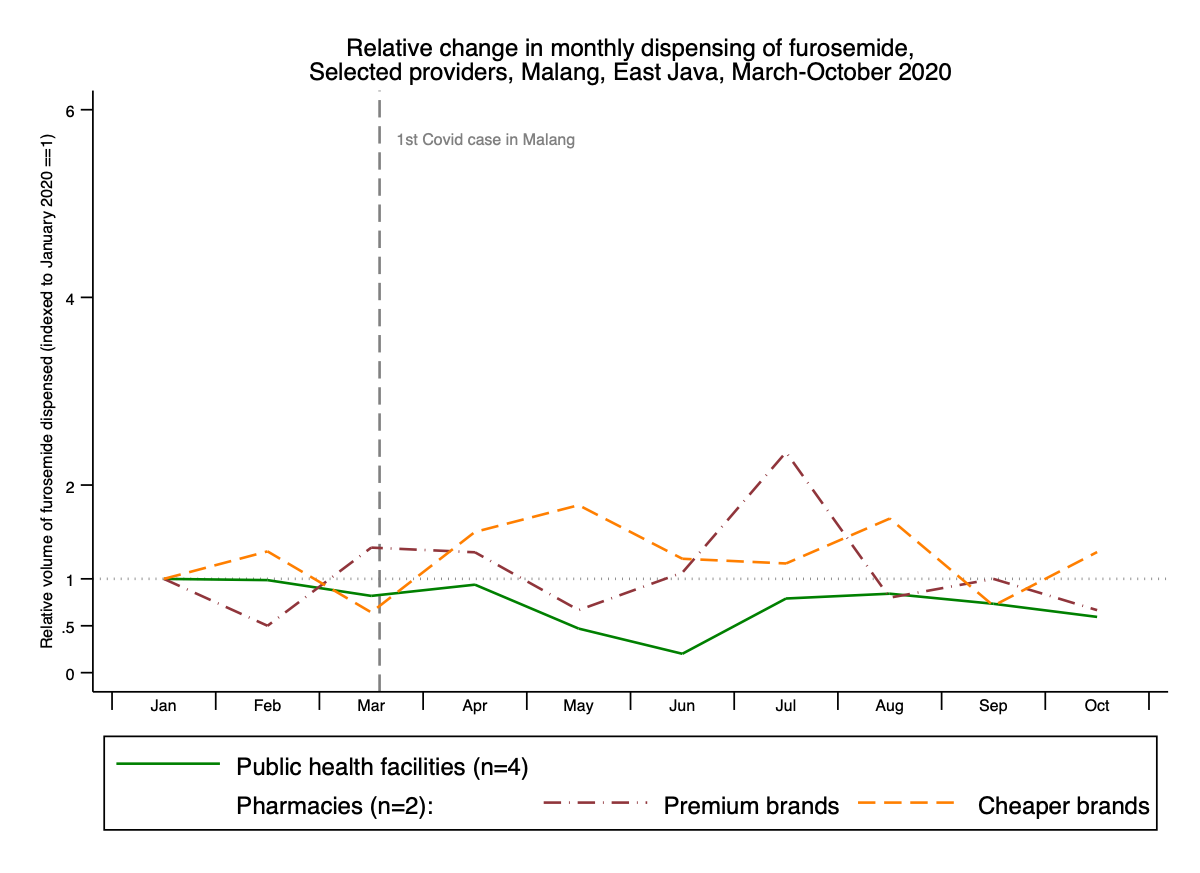

Supplement: Supplementary file 3 — Additional file 3: Fig. S1. C. Relative change in monthly dispensing of Furosemide, January - October 2020, by sector and price of Click. [file 40545_2022_509_MOESM3_ESM.png]

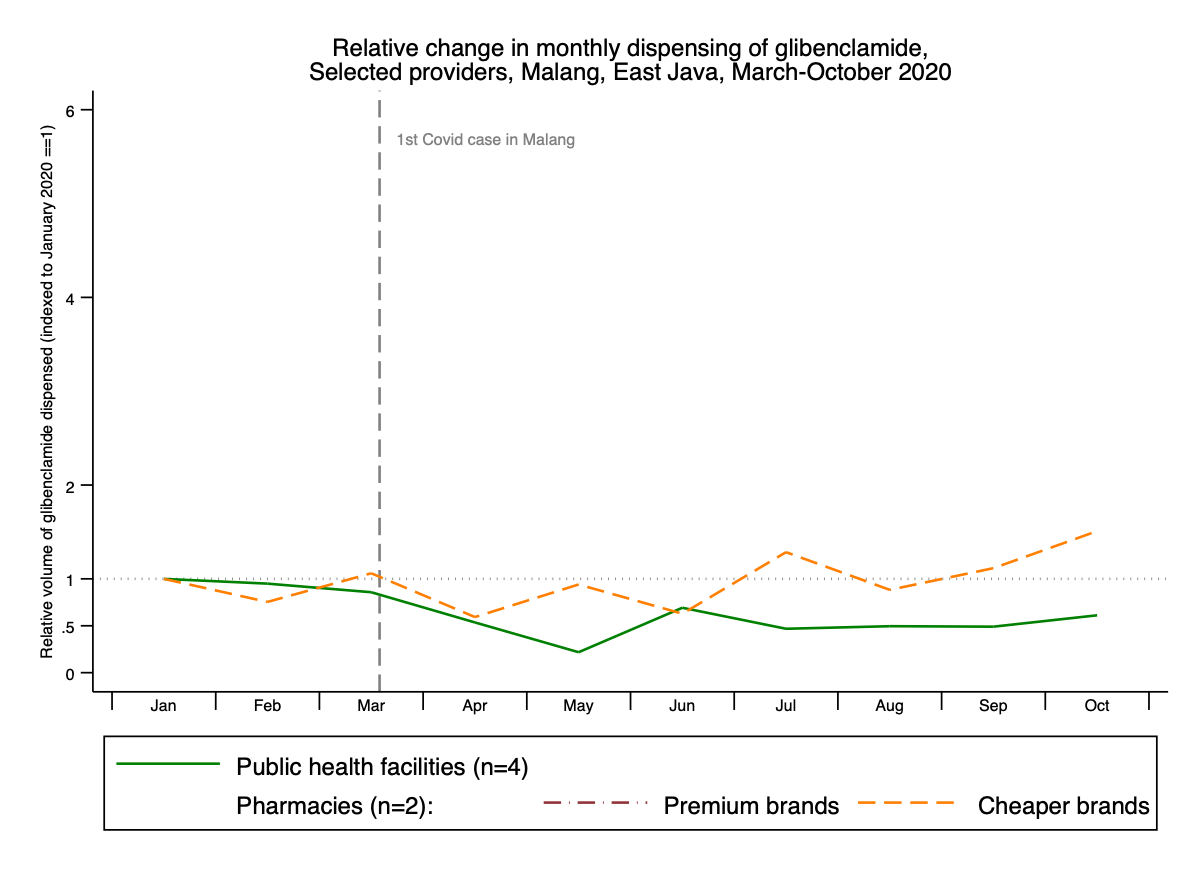

Supplement: Supplementary file 4 — Additional file 4: Fig. 1. D. Relative change in monthly dispensing of glibenclamide, January–October 2020, by sector and price of. [file 40545_2022_509_MOESM4_ESM.png]

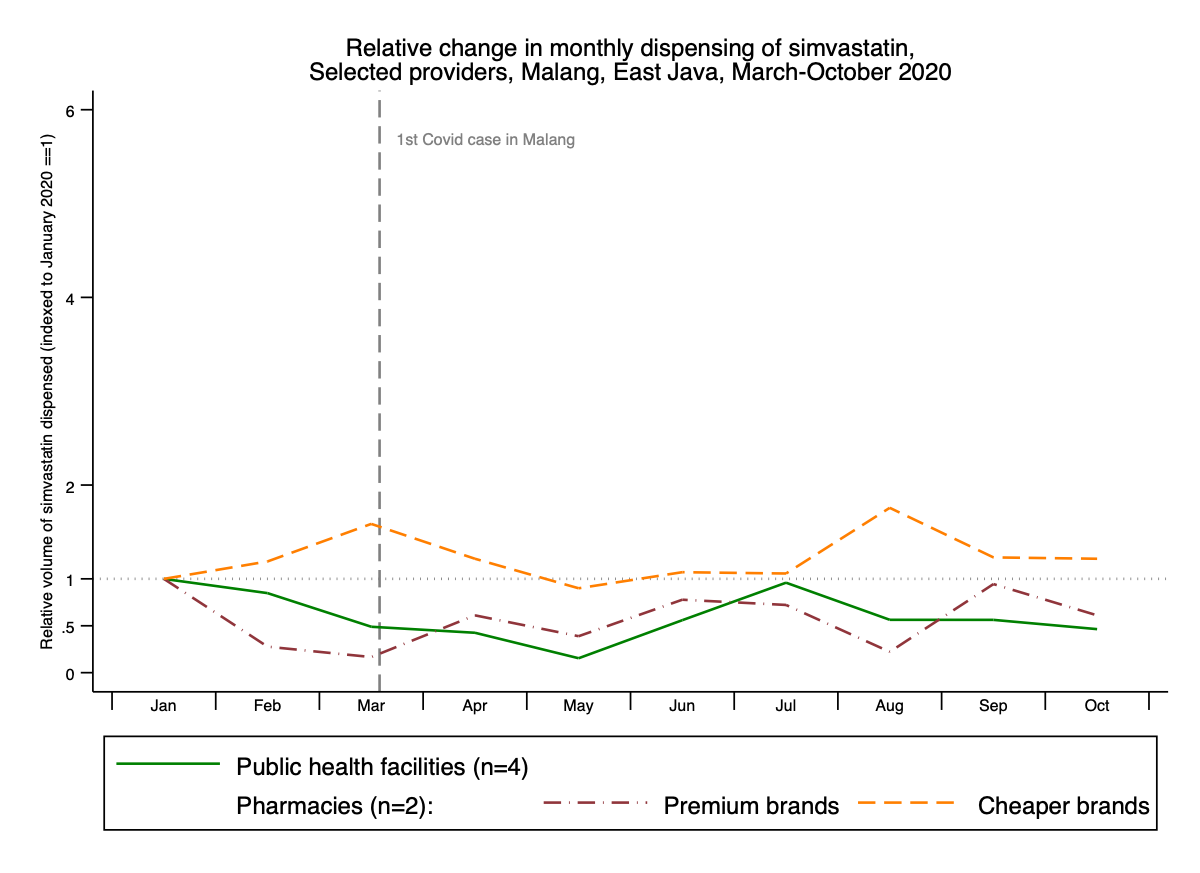

Supplement: Supplementary file 5 — Additional file 5: Fig. S1. E. Relative change in monthly dispensing of simvastatin, January–October 2020, by sector and price of medicine. [file 40545_2022_509_MOESM5_ESM.png]
